# Supplementary material for: Experimental interference of uncorrelated photons
Source: Sci Rep. 2019 Dec 5;9:18375. doi: 10.1038/s41598-019-54504-4 (PMC6895081; doi:10.1038/s41598-019-54504-4)
Supplement: Supplementary file 1 — Experimental interference of uncorrelated photons [file 41598_2019_54504_MOESM1_ESM.docx]

Supplementary information:

**Experimental interference of uncorrelated photons**

Heonoh Kim^1^, Osung Kwon^2,†^ & Han Seb Moon^1,*^

^1^Department of Physics, Pusan National University, Geumjeong-Gu, Busan 46241, South Korea

^2^Affiliated Institute of Electronics and Telecommunications Research Institute, Daejeon 34044, South Korea

^†^E-mail: oskwon@nsr.re.kr

^*^Corresponding author: hsmoon@pusan.ac.kr

**Supplementary Note 1: Generation of two-photon states with different internal/external degrees of freedom.**

Here, we consider the more general cases of generating two kinds of two-photon states at the two interferometer arms after photon passage through the 50:50 beamsplitter (BS). If we consider only two input photons regardless of whether the two photons have different ***internal degrees of freedom***, such as the orthogonal polarizations or different center frequencies, as shown in Supplementary Figure 1, the two-photon superposed states are generated with equal probability at the two BS-output ports 1 and 2 [1-5]. When the two photons enter together into one of the BS-input ports with orthogonal polarizations *H* and *V*, the polarization-entangled state, $\left| \left. \Psi\right\rangle\right.=1/\surd2\left( \left| \left. H \right\rangle\right._{1}\left| \left. V \right\rangle\right._{2}+\left| \left. V \right\rangle\right._{1}\left| \left. H \right\rangle\right._{2} \right)$, and the path-entangled state with orthogonally polarized photons, $\left| \left. \Psi\right\rangle\right.=1/\surd2\left( \left| \left. H,V \right\rangle\right._{1}\left| \left. 0 \right\rangle\right._{2}+\left| \left. 0 \right\rangle\right._{1}\left| \left. H,V \right\rangle\right._{2} \right)$, are coherently generated with equal probability in the two paths 1 and 2, depending on whether the two photons are split into two different spatial modes or the two photons propagate together along the same path (see Supplementary Figure 1(a,b)). Similarly, two different-frequency photons $\omega_{1}$ and $\omega_{2}$ generate the frequency-entangled state, $\left| \left. \Psi\right\rangle\right.=1/\surd2\left( \left| \left. \omega_{1} \right\rangle\right._{1}\left| \left. \omega_{2} \right\rangle\right._{2}+\left| \left. \omega_{2} \right\rangle\right._{1}\left| \left. \omega_{1} \right\rangle\right._{2} \right)$, and the dichromatic path-entangled state with different-frequency photons, $\left| \left. \Psi\right\rangle\right.=1/\surd2\left( \left| \left. \omega_{1},\omega_{2} \right\rangle\right._{1}\left| \left. 0 \right\rangle\right._{2}+\left| \left. 0 \right\rangle\right._{1}\left| \left. \omega_{1},\omega_{2} \right\rangle\right._{2} \right)$ (see Supplementary Figures 1(c,d)).

Supplementary Figure 2 illustrates the generation of two-photon states with different ***external degrees of freedom***, such as the spatial modes of the two input photons and the temporal separation between the two photons arriving at the two BS-input ports [4-6]. In the case that two identical photons are incident on the BS from two opposite input ports, the two photons always exit together through the same output port 1 or 2 of the BS with equal probability, as shown in Supplementary Figure 2(b), this phenomenon is the well-known Hong-Ou-Mandel (HOM) interference effect [7]. As a result, the path-entangled state, $\left| \left. \Psi\right\rangle\right.=1/\surd2\left( \left| \left. 2 \right\rangle\right._{1}\left| \left. 0 \right\rangle\right._{2}+\left| \left. 0 \right\rangle\right._{1}\left| \left. 2 \right\rangle\right._{2} \right)$, is generated in the two BS-output spatial modes 1 and 2. On the other hand, if the two photons arrive at the BS with a large time interval relative to the coherence time, two kinds of two-photon states are generated after the photons pass through the BS, as shown in Supplementary Figures 2(c) and 2(d) [5]. Moreover, in this type of two-photon state, the two individual photons do not have to possess the same polarization and frequency. The superposed state of two photons that are separately in the two spatial modes, as shown in Supplementary Figures 1(a), 1(c), and 2(c), result in a phase-insensitive HOM-type two-photon interference regardless of different internal/external degrees of freedom between the two photons [5]. On the other hand, the path-entangled state composed of two photons with different degrees of freedom reveals a highly phase-sensitive two-photon interference fringe [5,8].


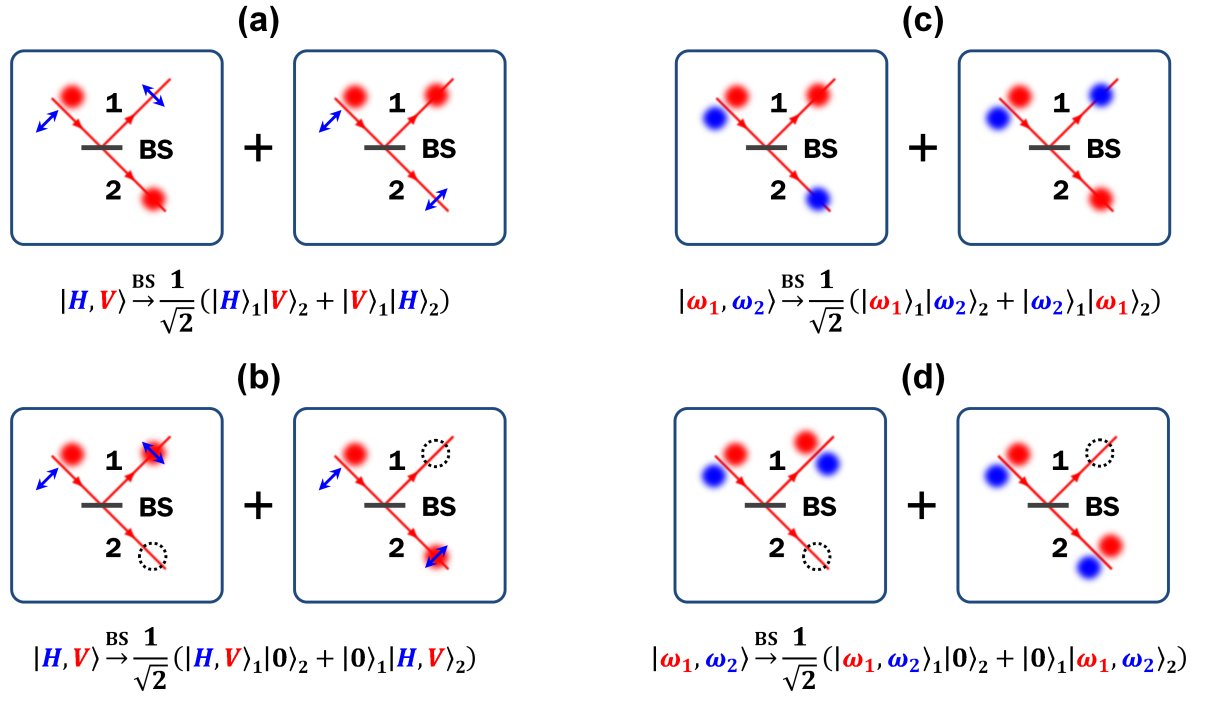


**Supplementary Figure 1. Generation of two two-photon states with different *internal* degrees of freedom**. The two-photon states are generated with (**a**,**b**) two orthogonally polarized photons and (**c**,**d**) two different-frequency photons. After the photons pass through the beamsplitter (BS), two kinds of two-photon states are generated in the two spatial modes 1 and 2.


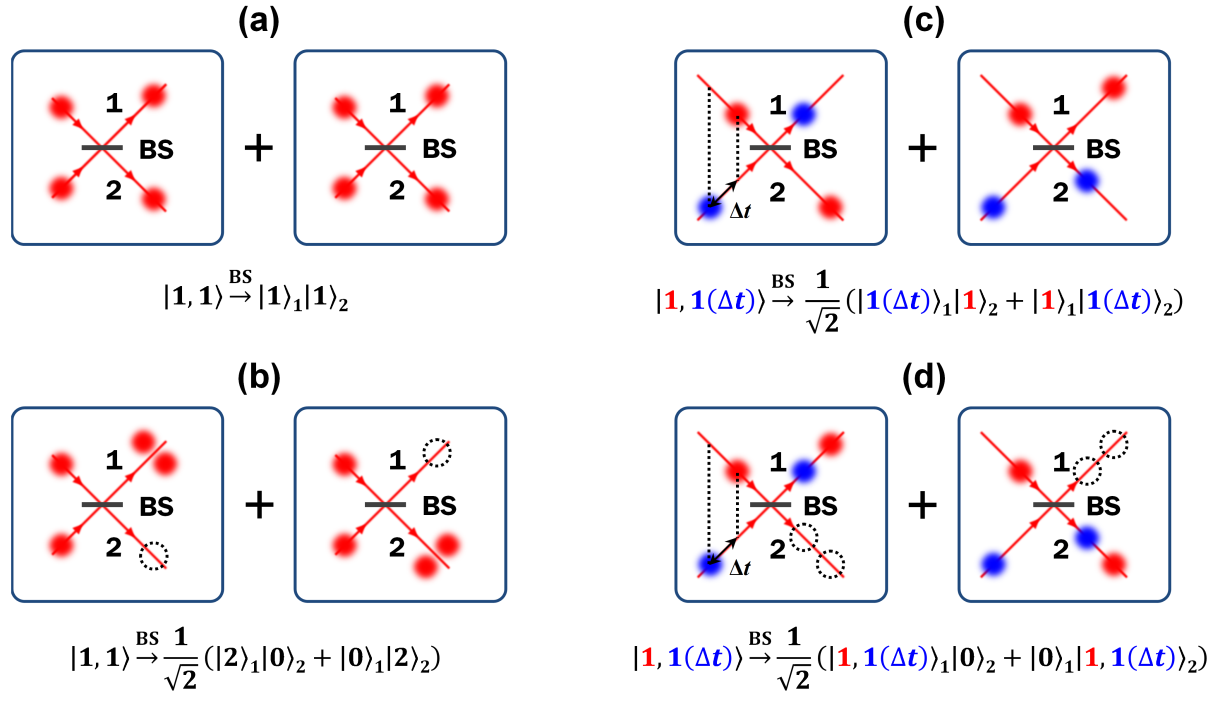


**Supplementary Figure 2. Generation of two two-photon states with different *external* degrees of freedom**. The two-photon states are generated with (**a**,**b**) two identical photons entering the beamsplitter (BS) from two opposite input ports and (**c**,**d**) two temporally well-separated photons in the two input ports. In the case of **a** and **b**, the two two-photon amplitudes corresponding to **a** disappear due to destructive interference, and therefore, only the two two-photon amplitudes corresponding to **b** remain.

**Supplementary Note 2: Time-delayed coincidence measurement of successive electrical signals from one single-photon detector.**

Recently, the method of the time-delayed coincidence measurement of successive electrical signals from one single-photon detector (SPD), which does not resolve more than two input photons, has been introduced to observe the HOM-type interference of two photons from weak coherent pulses [9]. The application of this measurement technique is possible when the two input photons are temporally well-separated relative to the dead time of the SPD used in the experiment. In our experiment, the observation of the two-photon interference fringe with only one SPD was carried out using a continuous-mode weak incoherent light source.


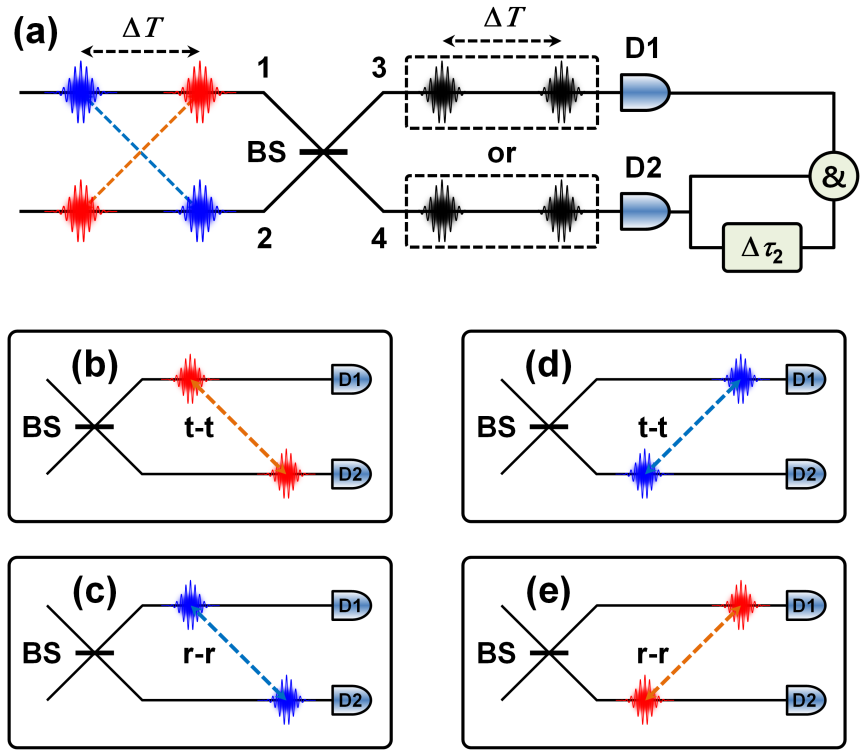


**Supplementary Figure 3. Observation of two-photon interference with one single-photon detector.** (**a**) Two-photon interference of temporally well-separated photons and the time-delayed coincidence measurement of successive electrical signals from one single-photon detector. (**b**,**c**) and (**d**,**e**) correspond to the two indistinguishable two-photon amplitudes after photon passage through the beamsplitter (BS).

Supplementary Figure 3(a) shows the two-photon interference between two two-photon amplitudes composed of two temporally well-separated pairwise photons with the use of time-delayed coincidence measurement employing electrical delay line (${\Delta\tau}_{2}$) after only one SPD D2. The coincidence measurement with two detectors D1 and D2(${\Delta\tau}_{2}$) can be used to compare the measurement result. Here, let us consider that two photons are incident on the 50:50 BS from two input ports 1 and 2 with a temporal delay $\Delta T$, which has to be longer than the dead time of the SPD. In this case, the two pairwise input photons are in a superposed state $1/\surd2\left[ a_{1}^{\dagger}a_{2}^{\dagger}\left( \Delta T \right)+a_{2}^{\dagger}a_{1}^{\dagger}\left( \Delta T \right) \right]\left| \left. 0,0 \right\rangle\right.$, where $a_{i}^{\dagger}$ denotes the photon- creation operator from vacuum state $\left| \left. 0 \right\rangle\right.$ of spatial modes 1 and 2. This situation can easily arise in the experimental setup shown in Fig. 1 (in the main text) when the two input photons are temporally well-separated. If there is no time difference between two amplitudes $a_{1}^{\dagger}a_{2}^{\dagger}\left( \Delta T \right)$ and $a_{2}^{\dagger}a_{1}^{\dagger}\left( \Delta T \right)$, then the two input photons always exit sequentially from the same output port 3 or 4 with 50% probability due to two-photon interference, as depicted in Supplementary Figure 3(a). Supplementary Figures 3(b) and 3(c) show the two-photon Feynman diagrams for indistinguishable amplitudes, the transmission-transmission (t-t) of the $a_{1}^{\dagger}a_{2}^{\dagger}\left( \Delta T \right)$ amplitude and reflection-reflection (r-r) of the $a_{2}^{\dagger}a_{1}^{\dagger}\left( \Delta T \right)$ amplitude, after passing through the BS. Similarly, Supplementary Figures 3(d) and 3(e) represent the t-t of $a_{2}^{\dagger}a_{1}^{\dagger}\left( \Delta T \right)$ and the r-r of $a_{1}^{\dagger}a_{2}^{\dagger}\left( \Delta T \right)$. The two-photon amplitudes $a_{4}^{\dagger}a_{3}^{\dagger}\left( \Delta T \right)$ or $a_{3}^{\dagger}a_{4}^{\dagger}\left( \Delta T \right)$ contributing to the time-delayed coincidence detection with D1&D2(${\Delta\tau}_{2}$) or D1(${\Delta\tau}_{1}$)&D2 are indistinguishable; therefore, these pairwise amplitudes destructively interfere with each other. As a result, the two photons arrive at the same detector D1 or D2 with a long time interval relative to the dead time of the SPD ($\Delta T\gg t_{d}$). It is interesting that the temporally well-separated pairwise two-photon state shows the same interferometric feature as in the case when the two single photons are incident on the BS simultaneously. With the electrical delay ${\Delta\tau}_{2}=\Delta T$ after the SPD D2, as shown in Supplementary Figure 3(a), the two-photon interference fringe can be observed with only one SPD D2.

**References**

1. Ou, Z. Y., Zou, X. Y., Wang, L. J. & Mandel, L. Experiment on nonclassical fourth-order interference. *Phys. Rev. A* **42**, 2957-2965 (1990).

2. Rarity, J. G. *et al*. Two-photon interference in a Mach-Zehnder interferometer. *Phys. Rev. Lett*. **65**, 1348-1351 (1990).

3. Larchuk, T. S. *et al*. Interfering entangled photons of different colors. *Phys. Rev. Lett*. **70**, 1603-1606 (1993).

4. Shih, Y. H., Sergienko, A. V., Rubirin, M. H., Kiess, T. E. & Alley, C. O. Two-photon interference in a standard Mach-Zehnder interferometer. *Phys. Rev. A* **49**, 4243-4246 (1994).

5. Kim, H., Lee, S. M. & Moon, H. S. Two-photon interference of temporally separated photons*. Sci. Rep*. **6**, 34805 (2016).

6. Jin, X. -M. *et al*. Sequential path entanglement for quantum metrology. *Sci. Rep.* **3**, 1779 (2013).

7. Hong, C. K., Ou, Z. Y. & Mandel, L. Measurement of subpicosecond time intervals between two photons by interference. *Phys. Rev. Lett.* **59**, 2044-2046 (1987).

8. Park, J., Kim, H. & Moon, H. S. Two-photon interferences of nondegenerate photon pairs from Doppler-broadened atomic ensemble. *Opt. Express* **25**, 32064-32073 (2017).

9. Kim, H., Lee, S. M., Kwon, O. & Moon, H. S. Observation of two-photon interference effect with a single non-photon-number resolving detector. *Opt. Lett.* **42**, 2443-2446 (2017).
